# Screening for RV Dysfunction Using Smartphone ECG Analysis App: Validation Study with Acute Pulmonary Embolism Patients

**DOI:** 10.3390/jcm13164792

**Published:** 2024-08-14

**Authors:** Yoo Jin Choi, Min Ji Park, Youngjin Cho, Joonghee Kim, Eunkyoung Lee, Dahyeon Son, Seo-Yoon Kim, Moon Seung Soh

**Affiliations:** 1 Department of Emergency Medicine, Ajou University School of Medicine, 164, World cup-ro, Yeongtong-gu, Suwon-si 16499, Gyeonggi-do, Republic of Korea; choiyj0729@naver.com (Y.J.C.); parkmjem@gmail.com (M.J.P.); seoyoon426@naver.com (S.-Y.K.); 2 Cardiovascular Center, Department of Internal Medicine, Seoul National University Bundang Hospital, 82, Gumi-ro 173 Beon-gil, Bundang-gu, Seongnam-si 13620, Gyeonggi-do, Republic of Korea; cho_y@snubh.org; 3 ARPI Inc., Room 12 Startup Incubation Center, 172, Dolma-ro, Bundang-gu, Seongnam-si 13605, Gyeonggi-do, Republic of Korea; joonghee@snubh.org (J.K.); eunkyoung0418@gmail.com (E.L.); sdh3130@gmail.com (D.S.); 4 Department of Emergency Medicine, Seoul National University Bundang Hospital, 82, 166 Gumi-ro 173 Beon-gil, Bundang-gu, Seongnam-si 13620, Gyeonggi-do, Republic of Korea; 5 Department of Cardiology, Ajou University School of Medicine, 164, World cup-ro, Yeongtong-gu, Suwon-si 16499, Gyeonggi-do, Republic of Korea

**Keywords:** RV dysfunction, pulmonary embolism, digital biomarkers, ECG analysis application, emergency department

## Abstract

**Background**: Acute pulmonary embolism (PE) is a critical condition where the timely and accurate assessment of right ventricular (RV) dysfunction is important for patient management. Given the limited availability of echocardiography in emergency departments (EDs), an artificial intelligence (AI) application that can identify RV dysfunction from electrocardiograms (ECGs) could improve the treatment of acute PE. **Methods**: This retrospective study analyzed adult acute PE patients in an ED from January 2021 to December 2023. We evaluated a smartphone application which analyzes printed ECGs to generate digital biomarkers for various conditions, including RV dysfunction (QCG-RVDys). The biomarker’s performance was compared with that of cardiologists and emergency physicians. **Results**: Among 116 included patients, 35 (30.2%) were diagnosed with RV dysfunction. The QCG-RVDys score demonstrated significant effectiveness in identifying RV dysfunction, with a receiver operating characteristic–area under the curve (AUC) of 0.895 (95% CI, 0.829–0.960), surpassing traditional biomarkers such as Troponin I (AUC: 0.692, 95% CI: 0.536–0.847) and ProBNP (AUC: 0.655, 95% CI: 0.532–0.778). Binarized based on the Youden Index, QCG-RVDys achieved an AUC of 0.845 (95% CI: 0.778–0.911), with a sensitivity, specificity, positive predictive value (PPV), and negative predictive value (NPV) of 91.2% (95% CI: 82.4–100%), 77.8% (95% CI: 69.1–86.4%), 63.3% (95% CI: 54.4–73.9%), and 95.5% (95% CI: 90.8–100%), respectively, significantly outperforming all the expert clinicians, with their AUCs ranging from 0.628 to 0.683. **Conclusions**: The application demonstrates promise in rapidly assessing RV dysfunction in acute PE patients. Its high NPV could streamline patient management, potentially reducing the reliance on echocardiography in emergency settings.

## 1. Introduction

Acute pulmonary thromboembolism (PE) is a serious emergency condition that can be life-threatening. Screening right ventricular (RV) dysfunction is crucial in the management of acute PE [1], as it significantly impacts patient outcomes. The RV’s response to increased afterload can result in myocardial ischemia and heart failure, increasing the risk of hemodynamic collapse and mortality [2]. Therefore, the timely and accurate detection of RV dysfunction is vital for risk stratification and for guiding treatment in acute PE.

Echocardiography is the standard tool for evaluating RV function, providing detailed hemodynamic information [3,4,5]. However, the fast-paced environment of the emergency department (ED) requires faster and simpler diagnostic methods, and in that regard, echocardiography may be limited in its use in the ED due to its reliance on skilled operators and the availability of equipment.

One potential solution to address this issue is to utilize electrocardiograms (ECGs). Recognizing RV strain patterns such as new right-axis deviations, the S1Q3T3 pattern, or ST-segment depressions, with T-wave inversions in leads V1 to V3 and leads II, III, and aVF from ECGs, can facilitate the evaluation of RV dysfunction [6,7]. However, these methods have limitations in accuracy, reducing their utility.

The integration of digital technology into medicine, and especially the use of artificial intelligence (AI) in acute care, offers new possibilities. AI solutions that analyze ECG data represent a shift toward more accessible and rapid cardiac assessments [8,9]. However, these applications typically require raw digital ECG data, which is impractical in real-world clinical settings where only printed ECG data are available.

To bridge this gap, we developed ECG Buddy^TM^, a mobile application that generates ten digital biomarkers by analyzing images of printed ECGs. Previous studies have suggested this tool’s utility in various emergency situations, including suspected myocardial infarctions and severe hyperkalemia [10,11,12,13]. This study aims to evaluate the application’s capability in identifying RV dysfunction in ED patients with acute PE. Additionally, we will compare its performance to those of expert clinicians.

## 2. Methods

### 2.1. Study Design and Data Collection

This study analyzed adult patients (≥18 years) with acute PE presenting at the ED of a tertiary hospital (Ajou University Medical Center) from January 2021 to December 2023 to validate AI software (version number 1.0.4.X) initially developed using data from a different hospital (Seoul National University Bundang Hospital). Patients were excluded if they did not have an ECG within 72 h post-ED arrival or an echocardiogram from 72 h before to 72 h after the ECG. Because one of the standard diagnostic tests for PE is a contrast-enhanced chest computed tomography (CT) scan, which is also commonly used in research facilities, the patients included in this study had their PE diagnosed by contrast-enhanced chest CT scan [2]. Two board-certified emergency physicians manually reviewed electronic medical records (EMR) to determine eligibility and gather information on demographics, PE risk factors, vital signs, lab results, and heart rhythm. The ECG data were collected from the EMR by manually cropping the waveform areas of each ECG report to remove any identifying information. The institutional review board approved this study (IRB No.: AJOUIRB-DB-2024-177), waiving informed consent due to its retrospective design.

### 2.2. Assessment of RV Function

The presence of RV dysfunction was determined by a qualitative review of echocardiographic reports considering the presence of a D-shaped left ventricle, Mcconnell’s sign, or decreased fractional area change (FAC < 35%). The FAC was calculated as (end-diastolic area–end-systolic area)/end-diastolic area x 100 in the RV-focused view of each echocardiogram [5]. In addition, measurements of echocardiographic right ventricular systolic pressure (RVSP), an indicator of pulmonary hypertension, were collected and categorized into four groups: RVSP I (less than 35 mmHg), RVSP II (35–49 mmHg), RVSP III (50–64 mmHg), and RVSP IV (greater than 64 mmHg). RVSP was determined from the peak tricuspid regurgitant jet velocity (TR Vpeak), using the simplified Bernoulli equation and combining this value with an estimate of the right atrium (RA) pressure estimated from the inferior vena cava diameter and the respiratory changes in the subcostal view [RVSP = 4(TR Vpeak)^2^ + RA pressure].

### 2.3. ECG Analysis by AI Application

An AI smartphone application, named “ECG Buddy”, was used to analyze the ECG images. The application, approved by the Korean MFDS and freely available for download in Korean appstores, can analyze 12-lead ECGs by taking pictures of ECG images to produce 10 digital biomarkers, (Quantitatve ECG[QCG^®^] scores ranging from 0 to 100) for various emergencies and cardiac dysfunctions [14]. We analyzed the ECG capture images by first displaying them on a desktop monitor and taking pictures of the ECG images using the application. We recorded digital biomarkers for RV dysfunction (QCG-RVDys) and pulmonary hypertension (QCG-PHTN) for each ECG for evaluation.

### 2.4. Expert Analysis of ECGs

To establish a benchmark for the AI biomarkers, expert evaluations of the same ECGs were obtained from a group of two cardiologists and three emergency physicians, all blinded to the patients’ clinical information. The experts, all board-certified physicians with at least 8 years of clinical experiences, asked to review each ECG image freely without a time limit to determine whether the ECG exhibited RV dysfunction primarily using an S1Q3T3 pattern [15,16].

### 2.5. Statistical Analysis

The primary metric used to assess the performance of the biomarkers was receiver operating characteristic–area under the curve (ROC-AUC). We compared the ROC-AUC of QCG-RVDys for identifying RV dysfunction to those for Troponin I and proBNP using original measurements in a continuous scale. For comparison with the experts, we binarized the biomarker using the threshold that maximized its Youden index (binarized QCG-RVDys), as the experts were asked to give their opinion in a binary format (yes or no). The sensitivity, specificity, positive predictive value (PPV), and negative predictive value (NPV) of the biomarker were calculated. The ROC-AUC of QCG-PHTN in identifying moderate pulmonary hypertension, as determined by RVSP 50 mmHg or more, was calculated too; however, the performance of the biomarker was not compared to other measurements, as it was not the main study topic of this study, and there is no popular method for identifying the condition clinically. All data analyses were conducted using R software, version 4.1.0.

## 3. Results

**Figure 1:**
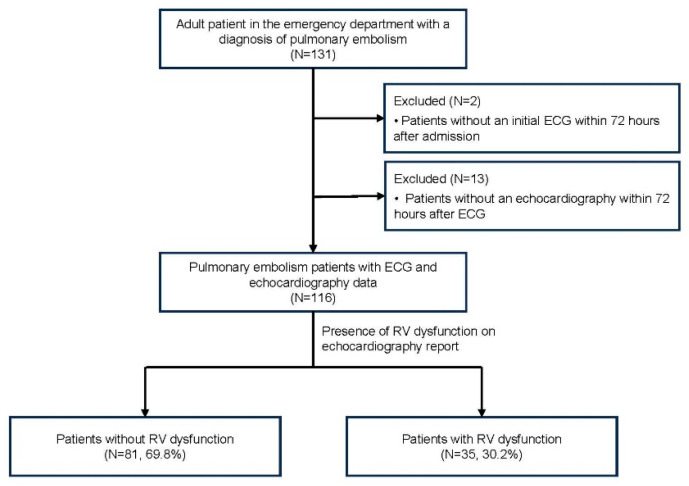
Patient selection flowchart.

From January 2021 to December 2023, 131 patients with acute PE were admitted to the emergency room. After the exclusion of fifteen patients lacking ECGs or echocardiography measurements meeting the eligibility criteria, a total of 116 patients were included in this study (Figure 1). Within the patient population, 35 patients were assessed as having RV dysfunction (RVD group, 30.2%) and 81 patients were assessed as not having RV dysfunction (No RVD group, 69.8%).

There were no statistically significant differences between the two groups in terms of previous risk factors, except for the number of patients with a history of deep vein thrombosis (No RVD: 0, RVD: 3, *p* = 0.042) (Table 1). Initial vital signs measured in the ED showed no significant differences. However, in blood tests, aspartate transaminase (AST; No RVD: 22.5 U/L vs. RVD: 32.0 U/L, *p* = 0.003), alanine transaminase (ALT; No RVD: 19.0 U/L vs. RVD: 30.0 U/L, *p* = 0.017), Troponin I (No RVD: 0.052 μg/mL vs. RVD: 0.176 μg/mL, *p* = 0.034), and proBNP (No RVD: 482.0 pg/mL vs. RVD: 1366.0 pg/mL, *p* = 0.024) were significantly higher in the RVD group. In addition, the RVSP group distribution was also significantly different (*p* < 0.001), and there was a trend of echocardiography being performed earlier in the RVD group (ED arrival to echocardiography, No RVD: 22.1 h vs. RVD: 16.1 h, *p*= 0.036).

**Figure 2:**
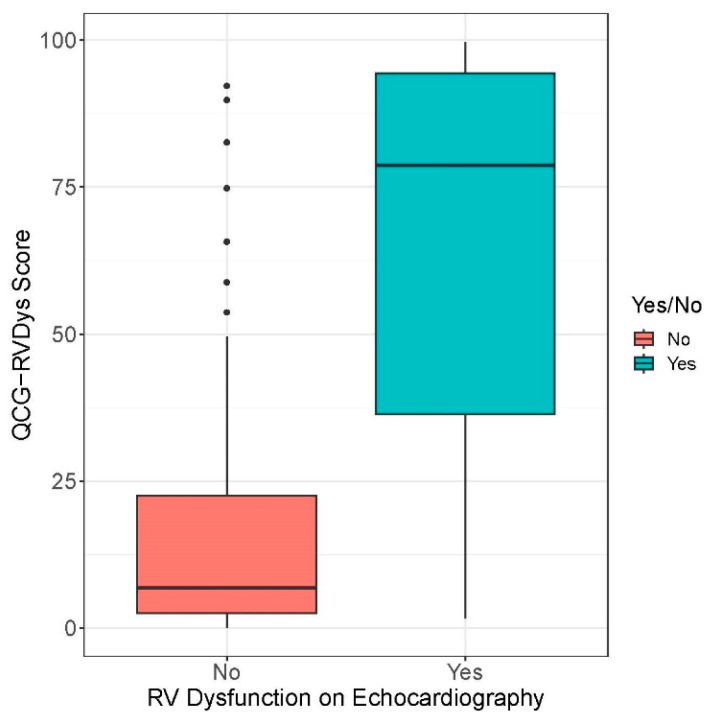
QCG score difference by RV dysfunction group.

The QCG-RVDys scores were significantly different between the No RVD group (6.8 [2.5–22.5]) and the RVD group (78.7 [35.7–94.9]), with a *p*-value of <0.001 (Figure 2, Table 2). The QCG-PHTN scores also showed significant differences across the RVSP groups I, II, III, and IV, demonstrating a clear correlation with increasing RVSP (*p* < 0.001, *p*-trend < 0.001, Table 2 and Figure S1).

**Figure 3:**
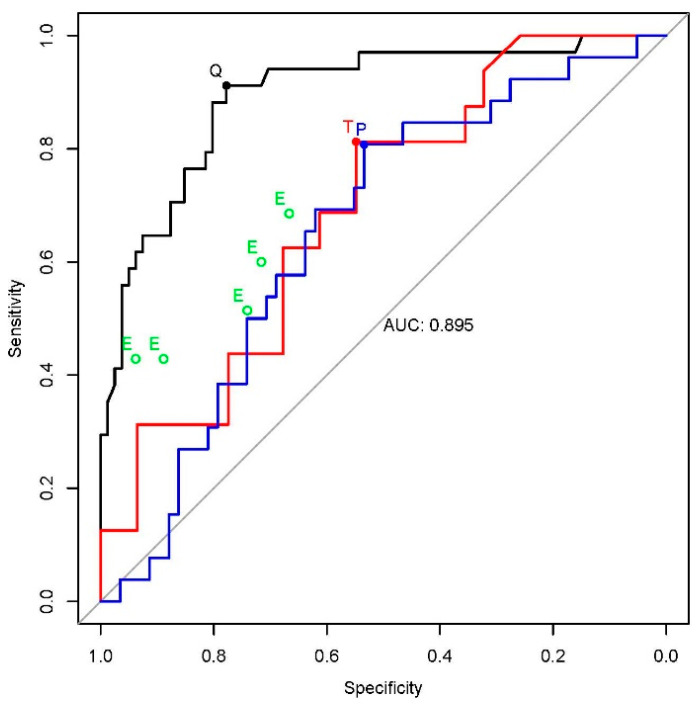
Performance of QCG-RVDys, Troponin I, ProBNP, and clinical experts on identifying RV dysfunction from ECGs (black line, QCG-RVDys; red line, Troponin I; blue line, ProBNP; green hollow dots, experts; black, red, and blue dots indicate binarized conditions).

The AUC-ROC for QCG-RVDys in diagnosing RV dysfunction was 0.895 (0.829–0.960), significantly higher than that for Troponin I, 0.692 (0.536–0.847), and ProBNP, 0.655 (0.532–0.778) (*p* = 0.046 and *p* = 0.001, respectively, Figure 3, Table 3). When the QCG-RVDys was binarized at a threshold of 24.65, based on the Youden index, the AUC was 0.845 (0.778–0.911), with a sensitivity, specificity, PPV, and NPV of 91.2% (82.4–100), 77.8% (69.1–86.4), 63.3% (54.4–73.9), and 95.5% (90.8–100), respectively. In comparison, the five experts showed AUCs ranging from 0.628 to 0.683, all statistically significantly lower than that of the binarized QCG-RVD. (Table S1).

The performance of QCG-PHTN in predicting elevated RVSP (RVSP groups III and IV; RVSP ≥ 50 mmHg) showed an ROC-AUC of 0.820 (0.728–0.912) (Table S2, Figure S2). At a threshold of 0.2590, the sensitivity, specificity, PPV, and NPV were 82.6% (65.2–95.7), 72.8% (64.1–81.5), 43.2% (34.7–54.1), and 94.4% (89.4–98.6), respectively.

To explore the potential factors influencing the QCG-RVDys score, we divided the patient population into two groups based on a threshold score of 24.65. Table S3 compares the characteristics of patients with QCG-RVDys scores above and below this threshold. We observed that patients with elevated QCG-RVDys scores (>24.65) were more likely to have higher levels of Troponin I and ProBNP, as well as higher RVSP in echocardiography, compared to those with lower scores. Additionally, differences in heart rhythm and laboratory measurements, such as white blood cell count and lactate levels, were noted between the two groups.

## 4. Discussion

This study showed that the digital ECG biomarker produced by a smartphone application can predict RV dysfunction with high accuracy in patients with acute PE using only printed ECGs without additional clinical information. We have also shown it can also predict pulmonary hypertension, too, expanding the utility of ECGs. This is the first study to assess critical cardiac functions in patients with acute PE using ECG AI, especially through a smartphone app, distinguishing it from similar research in the field.

Based on the results, smartphone-based ECG analysis software (version number 1.0.4.X) could be considered for use in treatment decisions for patients with acute PE in EDs. Assessing the right heart function, especially RV dysfunction and pulmonary hypertension, is crucial for determining the treatment plan for acute PE, alongside evaluating hemodynamic stability. This can rapidly guide decisions regarding thrombolytic therapy or surgical embolectomy in patients with RV dysfunction and hemodynamic instability [2,17,18]. According to our study, QCG-RVDys has a high NPV of 95.5 (90.8–100%), suggesting it could be useful in excluding the possibility of RV dysfunction in patients with newly diagnosed acute PE, for whom an immediate echocardiography is not feasible.

This utility can also be extended to the early diagnosis of massive PE. Patients with massive PE often present with nonspecific symptoms like dizziness, syncope, dyspnea, and chest pain, necessitating a broad differential diagnosis. In many EDs, when patients present with such symptoms, ECG is one of the first triage tests usually performed. Utilizing this, the early detection of RV dysfunction, often associated with massive PE, could prioritize the suspicion of PE, allowing for early diagnostic imaging, such as contrast chest CT, and hastening diagnosis [2]. Recently, studies have demonstrated the growing potential of AI models trained specifically to identify PE using ECG data [19,20,21,22,23,24]. In contrast, our model is designed to identify RV dysfunction, a critical characteristic of massive PE, which could serve as an early indicator in the diagnostic process. However, further studies are needed to determine which approach—the direct identification of PE or the detection of associated RV dysfunction—is more effective for early diagnosis and for improving patient outcomes.

Similarly, the evaluation of RV dysfunction could be expanded to other diseases, such as in acute respiratory distress syndrome and or pulmonale, where the assessment of RV strain is beneficial for establishing lung-protective ventilation strategies, balanced fluid therapy, the selection of vasopressors (minimizing impact on pulmonary vascular resistance), pulmonary vasodilation (e.g., NO, sildenafil), and effective monitoring strategies (echocardiography or hemodynamic measurement) [25,26,27,28,29]. Likewise, the early recognition of RV dysfunction in RV myocardial infarction through AI could aid safer decision making regarding early fluid therapy and vasodilator use. However, the efficacy and safety of the application in these specific clinical scenarios have not been evaluated, indicating a need for further research.

In the RVD group, the proportion of patients with RVSP >50 mmHg was higher, and the QCG-PHTN score increased with increasing RVSP, showing good utility. However, there was a wider distribution of QCG-PHTN scores in the high-RVSP group (especially >64 mmHg) compared to the relatively low-RVSP group, which can be explained by the fact that RVSP is not an absolute reflection of pulmonary hypertension and is influenced by the hemodynamics of the RV. In echocardiography, RVSP is mainly calculated from the tricuspid regurgitation maximal velocity (TR Vmax) and the collapsibility of the inferior vena cava [5]. In patients with RV dysfunction, the TR Vmax is also reduced, because the contractile force of the RV is reduced, which may cause this result.

The observed lower PPV of the QCG-RVDys score may be influenced by several patient characteristics that differ between those with scores above and below the threshold of 24.65. While higher Troponin I, ProBNP levels, and RVSP in the high-score group are consistent with the clinical features of PE, the increased prevalence of tachycardia and arrhythmias in this group could potentially influence the QCG-RVDys score. Although it is not yet clear whether these ECG abnormalities directly contribute to higher QCG-RVDys scores, their presence as common findings in emergency department patients may play a role. Further research is needed to clarify these potential confounding factors and refine the score’s predictive accuracy.

Handheld ultrasonography devices are increasingly being used to rapidly assess RV function at the bedside in patients with PE. These devices can detect key indicators of RV dysfunction, such as a dilated RV and D-sign, which are important in the evaluation of PE [30,31,32,33]. Integrating ECG Buddy™ with handheld ultrasonography could further improve PE diagnosis, and future studies should explore this combined approach to enhance early diagnosis and improve patient outcomes.

This study has several limitations. Firstly, it is a single-center, retrospective study with a small sample size, which may limit the generalizability of our findings. Therefore, a larger, multicenter, prospective study is needed. Second, we defined RV dysfunction based on echocardiography, which relies on the expertise of a trained examiner, and RVSP is not an absolute reflection of pulmonary hypertension, as it is dependent on RV dysfunction and cardiac physiology, and therefore needs to reflect a more appropriate endpoint. Finally, since this study was based on patients with already-diagnosed PE, a real-world point-of-care screening test would need to incorporate a variety of patient clinical conditions.

## 5. Conclusions

In conclusion, when predicting the RV dysfunction of digital ECG biomarkers in patients with acute PE using ECGs, smartphone software can more accurately assess the presence or absence of RV dysfunction compared to traditional methods by clinical specialists. Particularly, this smartphone software demonstrates a high negative predictive value, suggesting the potential to omit or delay costly and time-consuming echocardiography in patients of low risk.

## Figures and Tables

**Table 1:** Patient characteristics.

		Presence of RV Dysfunction		
		No (N = 81)	Yes (N = 35)	
Demographics	Age, years, median (IQR)	72.0 (50.0–81.0)	67.0 (45.5–77.0)	0.388
	Sex, male, N (%)	35 (43.2%)	14 (40.0%)	0.907
	Weight, kilograms, median (IQR)	61.3 (50.2–73.8)	55.8 (53.0–74.0)	0.946
	Height, centimeters, median (IQR)	160.0 (155.0–170.0)	162.0 (157.0–168.0)	0.781
Risk Factors of PTE	Diabetes Mellitus, N (%)	18 (22.2%)	5 (14.3%)	0.465
	Hypertension, N (%)	37 (45.7%)	12 (34.3%)	0.350
	Coronary artery occlusive disease, N (%)	7 (8.6%)	3 (8.6%)	1.000
	Cerebrovascular disease, N (%)	10 (12.3%)	1 (2.9%)	0.209
	Current smoker, N (%)	5 (6.2%)	3 (8.6%)	0.945
	Prolonged immobility (>1 week), N (%)	18 (22.2%)	8 (22.9%)	1.000
	Recent trauma or surgery (within 3 months), N (%)	16 (19.8%)	13 (37.1%)	0.080
	Active malignancy, N (%)	18 (22.2%)	3 (8.6%)	0.136
	Infectious disease (within 3 months), N (%)	19 (23.5%)	3 (8.6%)	0.105
	Hormone treatment, N (%)	2 (2.5%)	1 (2.9%)	1.000
	History of pulmonary thromboembolism, N (%)	10 (12.3%)	4 (11.4%)	1.000
	History of deep vein thrombosis, N (%)	0 (0.0%)	3 (8.6%)	0.042
Vital Signs	Systolic blood pressure, mean (SD)	130.0 (26.1)	129.4 (26.6)	0.915
	Diastolic blood pressure, mean (SD)	82.2 (17.7)	81.0 (15.1)	0.734
	Pulse rate, median (IQR)	99.0 (81.5–115.5)	101.0 (90.0–117.0)	0.150
	Respiratory rate, median (IQR)	20.0 (18.0–22.0)	21.0 (20.0–24.0)	0.092
Laboratory Measurements	White blood cell, 10^9^/L, median (IQR)	9.2 (7.4–13.1)	10.0 (7.3–13.5)	0.740
	Hemoglobin, g/dL, mean (SD)	12.1 (2.4)	12.4 (2.3)	0.486
	Aspartate transaminase, U/L, median (IQR)	22.5 (16.0–35.0)	32.0 (25.0–56.0)	0.003
	Alanine transaminase, U/L, median (IQR)	19.0 (12.0–34.0)	30.0 (18.0–60.5)	0.017
	Blood urea nitrogen, mg/dL, median (IQR)	14.9 (11.0–22.4)	14.3 (11.5–19.6)	0.551
	Creatinine, mg/dL, median (IQR)	0.9 (0.7–1.1)	0.8 (0.7–1.1)	0.440
	Troponin I, μg/mL, median (IQR)	0.052 (0.019–0.266)	0.176 (0.075–0.723)	0.034
	ProBNP, pg/mL, median (IQR)	482.0 (140.0–2400.0)	1366.0 (576.0–4733.0)	0.024
	D-dimer, mg/L, median (IQR)	3.6 (2.1–12.3)	4.6 (2.8–10.7)	0.599
	Lactate, mg/dL, median (IQR)	1.0 (0.8–2.3)	2.0 (1.1–2.3)	0.097
Heart rhythm (on ECG)				0.117
	Sinus Rhythm, N (%)	52 (64.2%)	14 (41.2%)	
	Sinus Tachycardia, N (%)	22 (27.2%)	16 (47.1%)	
	Atrial Fibrillation, N (%)	3 (3.7%)	1 (2.9%)	
	Multifocal Atrial Tachycardia, N (%)	2 (2.5%)	0 (0.0%)	
	Sinus Arrhythmia, N (%)	1 (1.2%)	0 (0.0%)	
	Atrial Rhythm, N (%)	0 (0.0%)	1 (2.9%)	
	Wandering Atrial Rhythm, N (%)	0 (0.0%)	1 (2.9%)	
	Undetermined Rhythm, N (%)	1 (1.2%)	1 (2.9%)	
Right Ventricular Systolic Pressure (RVSP)				<0.001
	RVSP I (<35 mmHg), N (%)	61 (75.3%)	4 (11.4%)	
	RVSP II (35–49 mmHg), N (%)	15 (18.5%)	12 (34.3%)	
	RVSP III (50–64 mmHg), N (%)	5 (6.2%)	10 (28.6%)	
	RVSP IV (>64 mmHg), N (%)	0 (0.0%)	9 (25.7%)	
Time of the test	ED arrival to ECG, hours, median (IQR)	1.0 (0.6–1.6)	0.8 (0.6–1.3)	0.388
	ED arrival to echocardiography, hours, median (IQR)	22.1 (16.0–35.6)	16.1 (4.8–27.5)	0.036
	ECG to echocardiography, hours, median (IQR)	20.4 (14.9–34.0)	13.8 (4.3–26.1)	0.045

RV, right ventricular; IQR, interquartile range; PTE, pulmonary thromboembolism; SD, standard deviation; BNP, blood natriuretic peptide; ECG, electrocardiogram; ED, emergency department.

**Table 2:** QCG score difference by RV dysfunction and across RVSP groups.

QCG Biomarker	Group	Biomarker Measurements, Median (IQR)	*p*
QCG-RVDys	RVD	6.8 (2.5–22.5)	<0.001 (for difference)
	No RVD	78.7 (35.7–94.9)	
QCG-PHTN	RVSP I (<35 mmHg)	8.1 (2.1–22.9)	<0.001 (for both difference and trend)
	RVSP II (35–49 mmHg)	21.7 (13.4–38.3)	
	RVSP III (50–64 mmHg)	41.6 (31.2–65.1)	
	RVSP IV (>64 mmHg)	49.2 (19.9–85.4)	

QCG, quantitative electrocardiography; IQR, interquartile range; RVDys, right ventricular dysfunction; RVD, right ventricular dysfunction; PHTN, pulmonary hypertension; RVSP, right ventricular systolic pressure.

**Table 3:** Performance of QCG and other biomarkers for prediction of RV dysfunction.

Biomarker	AUC	*p* for Difference	Sensitivity (95% CI)	Specificity (95% CI)	PPV (95% CI)	NPV (95% CI)	Threshold
QCG-RVDys (continuous scale)	0.895 (0.829–0.960)	-	91.2 (82.4–100)	77.8 (69.1–86.4)	63.3 (54.4–73.9)	95.5 (90.8–100)	24.65
Troponin I	0.692 (0.536–0.847)	0.046	81.2 (62.5–100)	54.8 (38.7–71)	48.1 (37.5–61.1)	85 (70.6–100)	0.0685 μg/mL
ProBNP	0.655 (0.532–0.778)	0.001	80.8 (65.4–96.2)	53.4 (39.7–65.5)	43.8 (35.6–52.5)	86.4 (75.8–96.3)	534.5 pg/mL

AUC, area under the curve; PPV, positive predictive value; CI, confidence interval; NPV, negative predictive value; QCG, quantitative electrocardiography; RVDys, right ventricular dysfunction; BNP, blood natriuretic peptide.

## Data Availability

The original contributions presented in this study are included in this article. Further inquiries can be directed to the corresponding author/s.

## Supplementary Materials

The following supporting information can be downloaded at https://www.mdpi.com/article/10.3390/jcm13164792/s1, Figure S1. QCG-PHTN score across RVSP groups (RVSP, right ventricular systolic pressure). Figure S2. Performance of QCG-PHTN on increased RVSP (RVSP ≥ 50 mmHg). Table S1. Performance of binarized QCG-RVDys and human experts on identifying RV dysfunction from ECG. Table S2. performance of binarized QCG-PHTN on increased RVSP (RVSP ≥ 50 mmHg). Table S3. Patient characteristics by RV dysfunction score.
