# Supplementary material for: Screening for RV Dysfunction Using Smartphone ECG Analysis App: Validation Study with Acute Pulmonary Embolism Patients
Source: J Clin Med. 2024 Aug 14;13(16):4792. doi: 10.3390/jcm13164792 (PMC11355826; doi:10.3390/jcm13164792)
Supplement: Supplementary file 1 [file jcm-13-04792-s001.zip › supplemtary materials.pdf]

Supplementary Materials

Supplementary Figure Legends

Fig. S1. QCG-PHTN score across RVSP groups (RVSP, right ventricular systolic pressure)

Fig. S2. Performance of QCG-PHTN on increased RVSP (RVSP >= 50mmHg)

Table S1. Performance of binarized QCG-RVDys and human experts on identifying RV dysfunction from ECG

Biomarker or expert	AUC (95% CI)	P for difference	Sensitivity (95% CI)	Specificity (95% CI)	PPV (95% CI)	NPV (95% CI)
QCG-RVDys (Binarized)	0.845 (0.778-0.911)	-	91.2 (82.4-100.0)	77.8 (69.1-86.4)	63.3 (54.4-73.9)	95.5 (90.8-100.0)
Expert #1	0.676 (0.583-0.770)	0.005	68.6 (51.4-82.9)	66.7 (56.8-76.5)	47.2 (38.2-57.2)	83.1 (75.8-90.5)
Expert #2	0.659 (0.569-0.749)	<0.001	42.9 (28.6-60.0)	88.9 (81.5-95.1)	62.5 (45.5-81.0)	78.2 (73.4-83.7)
Expert #3	0.628 (0.531-0.724)	<0.001	51.4 (34.3-68.6)	74.1 (64.2-84.0)	46.2 (34.2-60.0)	77.9 (71.9-84.6)
Expert #4	0.683 (0.596-0.771)	0.002	42.9 (28.6-60.0)	93.8 (87.7-98.8)	75.0 (57.7-92.9)	79.2 (74.7-84.1)
Expert #5	0.658 (0.562-0.754)	0.001	60.0 (45.7-77.1)	71.6 (62.9-81.5)	47.8 (37.5-60.0)	80.6 (74.3-87.8)

AUC, area under the curve; CI, confidence interval; PPV, positive predictive value; NPV, negative predictive value; QCG, quantitative electrocardiography; RVDys, right ventricular dysfunction;

Table S2. performance of binarized QCG-PHTN on increased RVSP (RVSP >= 50mmHg)

AUC	Sensitivity	Specificity	PPV	NPV	Threshold
0.820 (0.728-0.912)	82.6 (65.2-95.7)	72.8 (64.1-81.5)	43.2 (34.7-54.1)	94.4 (89.4-98.6)	0.259

AUC, area under the curve; PPV, positive predictive value; NPV, negative predictive value;

Table S3. Patient characteristics by RV dysfunction score

		Elevated RVDys score > 24.65		P
		No (N=66)	Yes (N=49)	
Demographics	Age, years (IQR)	67.0(41.0-79.0)	73.0(56.0-81.0)	0.183
	Sex, male (%)	29(43.9%)	19(38.8%)	0.716
	Weight, kilograms (IQR)	65.6(52.5-75.3)	55.8(51.9-67.0)	0.077
	Height, centimeters (IQR)	163.0(155.0-170.0)	160.0(155.0-168.0)	0.377
Risk Factors of PTE	DM	12(18.2%)	11(22.4%)	0.741
	HTN	25(37.9%)	23(46.9%)	0.434
	Coronary artery occlusive disease	2(3.0%)	8(16.3%)	0.030
	Cerebrovascular disease	6(9.1%)	5(10.2%)	1.000
	Current Smoker	6(9.1%)	1(2.0%)	0.242
	Prolonged immobility (>1 week)	15(22.7%)	11(22.4%)	1.000
	Recent trauma or surgery (within 3 months)	15(22.7%)	14(28.6%)	0.620
	Active malignancy	14(21.2%)	7(14.3%)	0.480
	Infectious disease (within 3 months)	16(24.2%)	5(10.2%)	0.092
	Hormone treatment	2(3.0%)	1(2.0%)	1.000
	History of pulmonary thromboembolism	6(9.1%)	8(16.3%)	0.376
	History of deep vein thrombosis	0(0.0%)	3(6.1%)	0.148
Vital Signs	Systolic blood Pressure	133.6(21.5	123.8(30.6	0.075
	Diastolic blood pressure	82.7(15.4	80.1(18.7	0.449
	Pulse rate	96.5(81.0-113.0)	104.0(88.0-119.0)	0.073
	Respiratory rate	20.0(18.0-24.0)	20.0(19.5-23.0)	0.757
Laboratory Measurements	White blood cell, unit	8.8(7.0-10.6)	11.4(7.6-14.6)	0.010
	Hemoglobin, Unit	12.1(2.4	12.3(2.4	0.696
	Aspartate transaminase	22.0(16.0-35.0)	31.0(22.0-56.0)	0.002
	Alanine transaminase	19.0(12.5-33.5)	24.0(16.0-59.0)	0.062
	Blood urea nitrogen	12.8(10.6-21.1)	16.4(12.6-21.2)	0.126
	Creatinine	0.8(0.7-1.1)	0.9(0.7-1.2)	0.452
	Troponin I	0.028(0.014-0.266)	0.158(0.057-0.431)	0.027
	ProBNP (blood natriuretic peptide)	368.0(104.0-854.0)	1596.0(719.0-4862.0)	<0.001
	D-dimer	3.3(1.9-7.1)	5.2(2.9-12.2)	0.082
	Lactate	1.0(0.7-1.7)	2.0(1.1-3.0)	0.002
Heart rhythm (on ECG)				0.022
	Sinus Rhythm	47(71.2%)	19(38.8%)	
	Sinus Tachycardia	15(22.7%)	23(46.9%)	
	Atrial Fibrillation	2(3.0%)	2(4.1%)	
	Multifocal Atrial Tachycardia	1(1.5%)	1(2.0%)	
	Sinus Arrhythmia	1(1.5%)	0(0.0%)	
	Atrial Rhythm	0(0.0%)	1(2.0%)	
	Wandering Atrial Rhythm	0(0.0%)	1(2.0%)	
	Undetermined Rhythm	0(0.0%)	2(4.1%)	
Echocardiography	Ejection Fraction, %	66.2(7.8	64.9(8.8	0.402
	Right Ventricular Systolic Pressure			<0.001
	<35	49(74.2%)	16(32.7%)	
	35-49	13(19.7%)	14(28.6%)	
	50-64	3(4.5%)	12(24.5%)	
	>64	1(1.5%)	7(14.3%)	
Time of the test	ED arrival to ECG, hours	1.1(0.6-1.6)	0.8(0.5-1.2)	0.083

	ED arrival to echocardiography, hours	22.0(14.1-36.4)	18.3(7.8-31.9)	0.411
	ECG to echocardiography, hours	19.6(13.3-34.8)	17.3(7.0-31.5)	0.478
Dispositions	Hospital Admission	61(92.4%)	47(95.9%)	0.703
	Survival Discharge (within 30 days)	62(93.9%)	42(85.7%)	0.245

RV, right ventricular; IQR, interquartile range; PTE, Pulmonary thromboembolism; SD, standard deviation; BNP, Blood natriuretic peptide; ECG, Electrocardiogram; ED, Emergency department.
