# Supplementary figures and images for: Screening for RV Dysfunction Using Smartphone ECG Analysis App: Validation Study with Acute Pulmonary Embolism Patients
Source: J Clin Med. 2024 Aug 14;13(16):4792. doi: 10.3390/jcm13164792 (PMC11355826; doi:10.3390/jcm13164792)

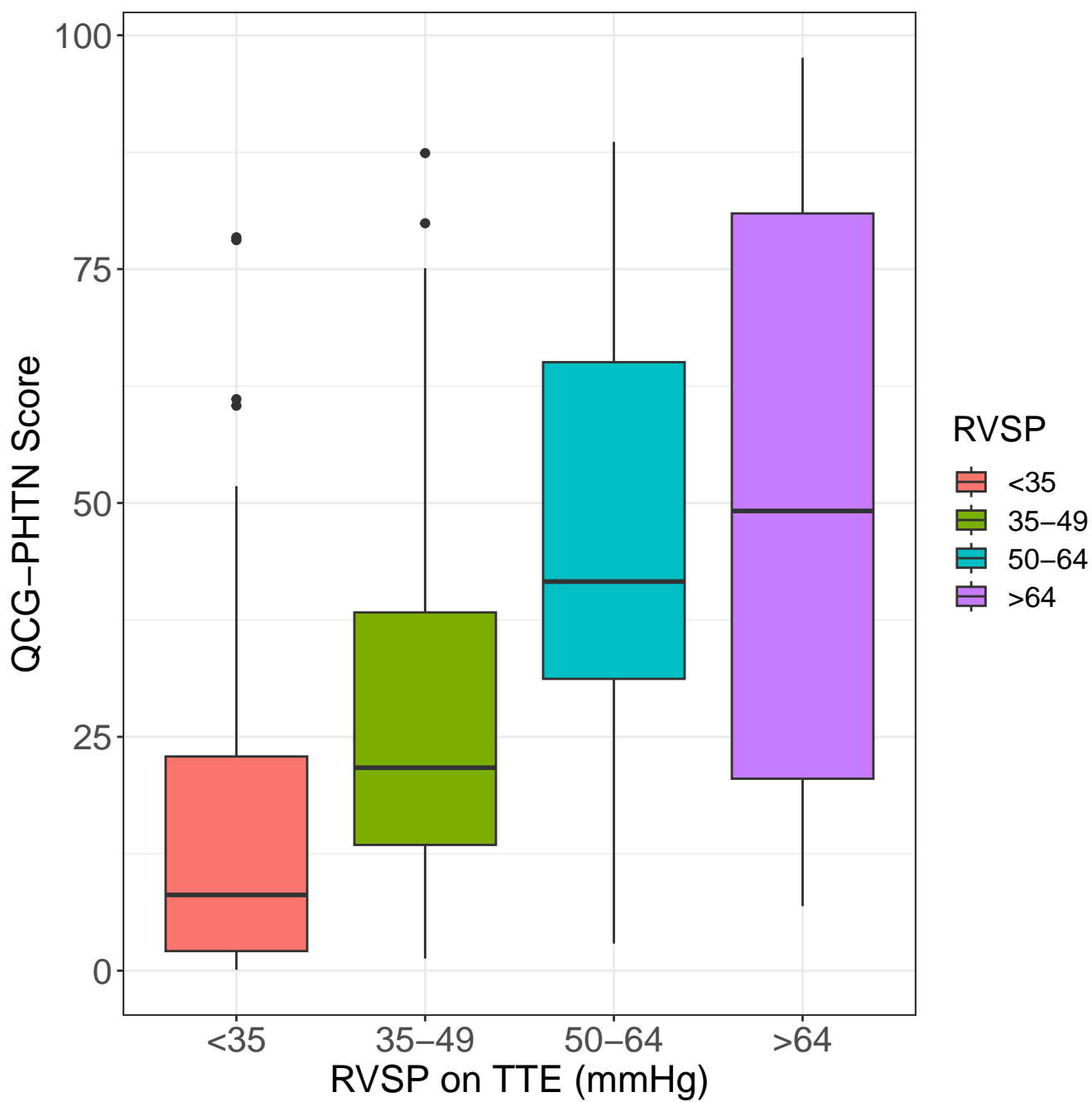

Supplement: Supplementary file 1 [file jcm-13-04792-s001.zip › Fig. S1.pdf]

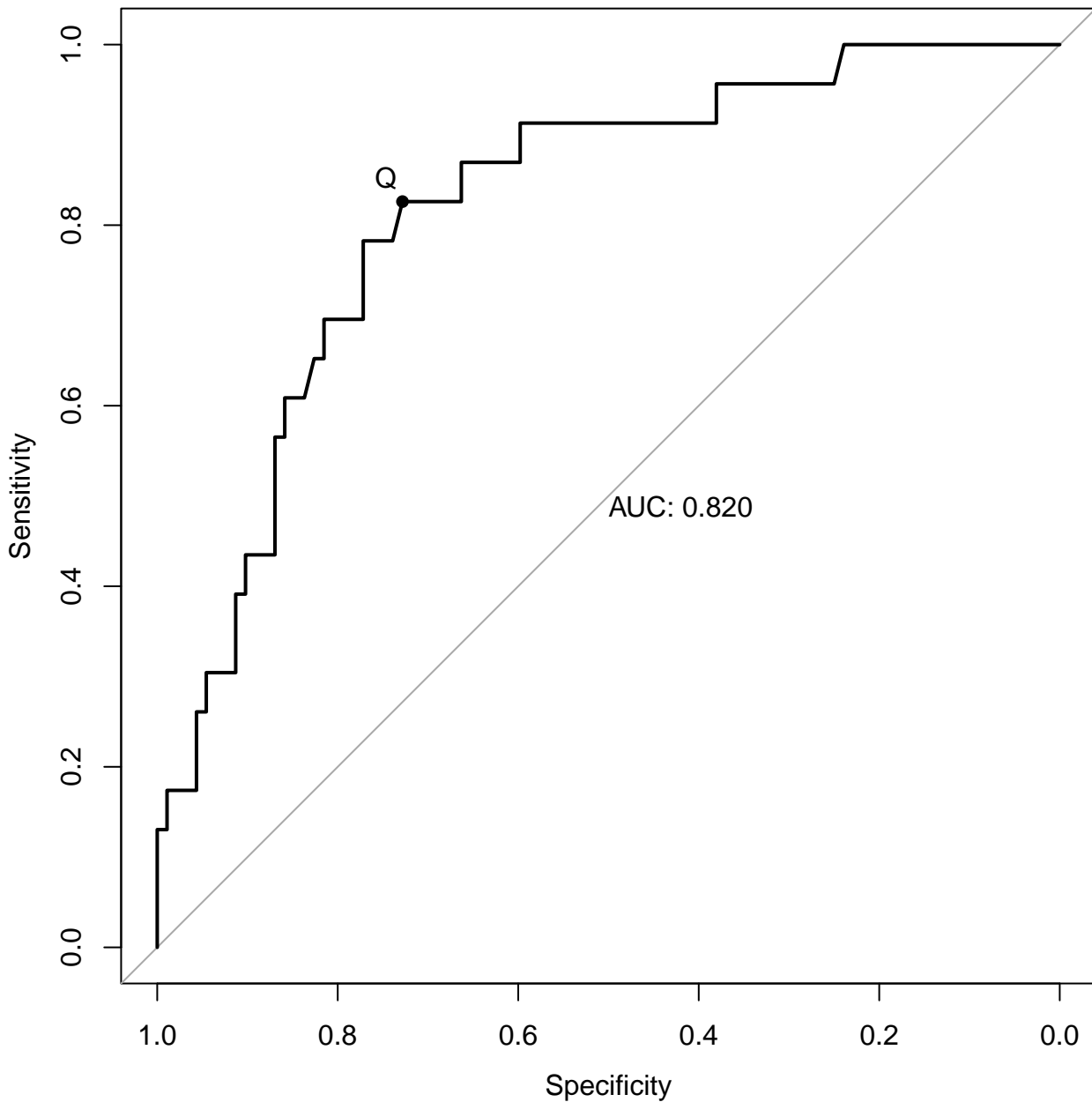

Supplement: Supplementary file 1 [file jcm-13-04792-s001.zip › Fig. S2.pdf]
